# 免疫检查点抑制剂相关心脏不良反应的临床诊治建议

**DOI:** 10.3779/j.issn.1009-3419.2019.10.04

**Published:** 2019-10-20

**Authors:** 潇潇 郭, 汉萍 王, 佳鑫 周, 炼 段, 玥 李, 晓燕 斯, 丽 张, 理刚 方, 力 张

**Affiliations:** 1 100730 北京，中国医学科学院，北京协和医学院，北京协和医院心内科 Department of Cardiology, Peking Union Medical College Hospital, Peking Union Medical College and Chinese Academy of Medical Sciences, Beijing 100730, China; 2 100730 北京，中国医学科学院，北京协和医学院，北京协和医院呼吸内科 Department of Respiratory Medicine, Peking Union Medical College Hospital, Peking Union Medical College and Chinese Academy of Medical Sciences, Beijing 100730, China; 3 100730 北京，中国医学科学院，北京协和医学院，北京协和医院风湿免疫科 Department of Rheumatism and Immunology, Peking Union Medical College Hospital, Peking Union Medical College and Chinese Academy of Medical Sciences, Beijing 100730, China; 4 100730 北京，中国医学科学院，北京协和医学院，北京协和医院内分泌科 Department of Endocrinology, Peking Union Medical College Hospital, Peking Union Medical College and Chinese Academy of Medical Sciences, Beijing 100730, China; 5 100730 北京，中国医学科学院，北京协和医学院，北京协和医院消化内科 Department of Digestive Medicine, Peking Union Medical College Hospital, Peking Union Medical College and Chinese Academy of Medical Sciences, Beijing 100730, China; 6 100730 北京，中国医学科学院，北京协和医学院，北京协和医院检验科 Department of Clinical Laboratory, Peking Union Medical College Hospital, Peking Union Medical College and Chinese Academy of Medical Sciences, Beijing 100730, China

**Keywords:** 免疫检查点抑制剂, 免疫相关不良反应, 心肌炎, 心血管毒副反应, Immune checkpoint inhibitor, Immunotherapy-related toxicities, Myocarditis, Cardiovascular toxicities

## Abstract

恶性肿瘤的免疫治疗已经成为肿瘤研究和治疗领域的热点，给晚期肿瘤患者带来了新的希望。免疫检查点分子程序性死亡蛋白-1和细胞毒性T淋巴细胞相关抗原4相关信号通路的激活可以抑制T淋巴细胞活化，从而阻断炎症反应。肿瘤细胞通过激活免疫检查点分子相关的信号通路实现免疫逃逸。而免疫检查点抑制剂能够唤醒T淋巴细胞，增强机体对肿瘤细胞的清除。但是免疫检查点抑制剂的作用并非肿瘤细胞特异，在杀灭肿瘤细胞的同时，会引起包括心血管系统在内的多系统副作用。我们将总结相关的心脏副反应并对如何防治给予建议。

## Abstract (en)

Immunotherapy of malignant tumors has become a hot spot in the field of cancer research and treatment, bringing new hope to patients with advanced cancer. Activation of molecular programmer death protein-1 and T lymphocyte-associated antigen 4-related signaling pathways at the immunological checkpoint can inhibit T lymphocyte activation and thereby block the inflammatory response. Tumor cells achieve immune escape by activating the molecular pathways associated with immune checkpoints. The immune checkpoint inhibitor can wake up T lymphocytes and enhance the body's clearance of tumor cells. However, the role of immune checkpoint inhibitors is not specific to tumor cells, and it can cause side effects of multiple systems including the cardiovascular system while killing tumor cells. We will summarize the relevant cardiac side effects and give advice on how to manage it.

免疫系统是人体监视肿瘤发生发展的一道系统屏障，肿瘤细胞通过改变自身生物学特征逃避免疫监视或通过削弱机体的免疫监视能力实现自我生长。近年来，机体免疫因素在肿瘤发生与治疗中的作用受到越来越多的关注。目前，肿瘤免疫检查点治疗已经成为肿瘤研究和治疗领域的热点，并不断取得令人瞩目的成绩，在黑色素瘤、非小细胞肺癌、结肠癌、肾细胞癌等肿瘤的治疗中尤其展示了非凡的应用前景。已经进入临床应用的免疫检查点分子主要分为两种。程序性死亡蛋白-1（programmed cell death protein-1, PD-1）在T淋巴细胞膜上表达并通过与肿瘤细胞高表达的程序性死亡分子配体1（programmed cell death ligand-1, PD-L1）结合，激活PD-1信号通路，使得T细胞功能受损。帕博利珠单抗（pembrolizumab）、纳武利尤单抗（nivolumab）和阿特珠单抗（Atezolizumab）针对这一信号通路阻断PD-1与PD-L1的结合，阻断负向调控信号通路，恢复T细胞的功能活性，从而增强机体对肿瘤的免疫杀伤作用。另一种分子是细胞毒性T淋巴细胞相关抗原4（cytotoxic T lymphocyte associated antigen 4, CTLA4），也是一种T细胞膜表面表达的抑制性受体，CTLA4被激动后产生抑制性信号，使T细胞的活化被抑制。伊匹木单抗（ipilimumab）可阻断CTLA-4与其活化信号的结合，去除肿瘤免疫抑制，调动特异性抗肿瘤反应。但是，随着肿瘤免疫检查点治疗药物的蓬勃发展，这些新型肿瘤治疗药物带来的各系统副反应也相继浮出水面；药物的临床安全使用面临挑战^[1, 2]^。现对免疫检查点抑制剂（immune checkpoint inhibitor, ICI）应用过程中所观察到的心脏不良反应作一综述，以提高临床医生对ICI不良反应的警惕性和应对能力。

## 1 ICI的心脏副作用

在ICI各系统副作用中，心脏系统的不良反应发生率较低。已报道的不良反应包括心肌病变（心肌炎为主）、心包积液、心律失常、急性冠脉综合征和瓣膜病变等^[3]^。根据以往的临床试验和回顾性研究，心脏不良事件虽然不常发生，但是具有高致死性的特点，其中心肌炎的致死率高达39.7%-50%^[4-6]^。

### 1.1 ICI相关心肌病变

#### 1.1.1 ICI相关心肌炎

心肌炎是ICI比较少见的心脏副作用之一，发生率在0.06%-3.8%，发生时间在首次用药后15 d-30 d。常呈急性或爆发性发作的特征，患者出现胸痛、活动时呼吸困难以及下肢水肿等临床症状，并在数天或1周-2周内迅速加重，甚至出现心源性休克或心脏骤停。实验室检查血肌钙蛋白轻至中度升高，但不符合急性心肌梗死的升降规律；血脑钠肽（brain natriuretic peptide, BNP）或N末端脑钠肽前体（NT-proBNP）进行性升高；心电图检查可见ST-T改变，部分导联R波消失Q波形成，甚至QRS波增宽，可能合并多种心律失常，如室性早搏、持续性室速等。超声心动图检查可见左室壁普遍或节段性运动减低，心腔多无明显扩大，左室（可能合并右室）射血分数减低，伴或不伴中重度瓣膜关闭不全。冠脉增强CT或冠状动脉造影无阻塞性冠状动脉疾病证据。心脏增强核磁共振（cardiac contrast-enhanced magnetic resonance, CMR）可见心肌水肿和/或心肌内延迟强化，左室射血分数轻-中度降低^[7]^；心内膜活检可见心肌细胞变性、坏死、纤维化，以及T淋巴细胞浸润，有个案报道可见多核巨细胞心肌浸润^[8, 9]^。

#### 1.1.2 ICI相关心肌病

接受ICI治疗的患者，如果出现乏力、进行性加重的呼吸困难和外周水肿，除了心肌炎的可能外，还需要考虑扩张型心肌病。两者在临床症状方面较难鉴别，但是心肌病的发生发展过程较为隐匿。此类患者实验室检查血肌钙蛋白基本正常，BNP或NT-proBNP明显升高。心电图也会有QRS波形改变和/或增宽，不特异的ST-T改变和各种心律失常；超声心动图可见节段性或弥漫的室壁运动减弱，左室腔内径正常或轻度增大，左室（可能合并右室）射血分数降低，可以合并中重度的主动脉瓣或二、三尖瓣关闭不全。冠脉增强CT或冠状动脉造影无阻塞性冠状动脉疾病证据。CMR多见心肌内延迟强化，无明显心肌水肿；心内膜活检病理可见心肌细胞变性、坏死、间质纤维化，但无炎性细胞浸润^[8]^。个别患者无明显心力衰竭的临床症状，但是超声心动图发现心脏收缩功能下降、左室变大或者尸检发现心脏增大、心肌内弥漫纤维化^[8, 9]^。

#### 1.1.3 应激性心肌病

这是另一类有个案报道的与ICI使用有关的心肌病^[10]^。临床症状与心肌炎较难鉴别，出现持续胸闷、胸痛，严重者进展为静息喘憋不能平卧。心电图可见多个相邻导联ST段抬高或压低，T波异常，可以合并多种房性及室性心律失常，甚至心脏骤停；生化检查肌钙蛋白正常或轻度升高，BNP或者NT-proBNP常明显升高；典型的超声心动图表现为节段性室壁运动减弱，以左室中下段及心尖部为著，而左室基底部心肌运动大致正常，整个左室收缩时呈“捕鱼篓形”，运动异常的室壁节段超过心外膜单支冠状动脉供血区域；冠脉增强CT或冠状动脉造影无阻塞性冠状动脉疾病证据。CMR可见心肌水肿但是通常无心肌延迟强化的表现。这类患者需要早期行冠状动脉评估并与急性冠脉综合征鉴别，另外超声心动图典型的形态学改变以及CMR和心内膜活检有助于与急性心肌炎鉴别。

上述三种心肌病变均为个案报道，其中心肌炎报道最多，后两种报道较少，也可能与临床对它们的认识不够有关。

### 1.2 ICI相关心包炎

多数表现为心包积液，中位发生时间在首次用药后30 d。患者出现进行性加重的活动后呼吸困难，心电图表现为QRS波低电压，胸片见烧瓶样心影增大，超声心动图可明确诊断。短期快速的心包积液增长会引起心包填塞，有研究^[5]^统计ICI相关心包积液中81%会造成血流动力学异常，需要急诊心包穿刺。

### 1.3 ICI相关心律失常

在ICI相关心律失常中，以房性心律失常的发生率最高，如窦性心动过速、房性早搏、房性心动过速、房扑甚至房颤；房性心律失常更多的与患者的心脏外情况有关，比如年龄较大、低氧、感染、肺部病变或者ICI相关的内分泌异常如甲状腺功能异常^[5]^。相对发生率较低的心律失常如包括频发室性早博、非持续性室速、尖端扭转室速以及房室传导阻滞甚至心脏骤停，大多伴发心肌病变尤其是心肌炎，预后更差。临床上如果出现心悸、头晕、黑矇或意识丧失，要考虑心律失常可能，如果心电图或24 h动态心电图出现上述室性心律失常，应该立刻开展对心脏情况的全面评估。

### 1.4 ICI相关心肌缺血

已有报道应用ICI的患者可出现稳定性心绞痛或急性冠脉综合征，甚至需要急诊行冠状动脉介入治疗，具体发生率尚不明确。动物实验^[10]^观察到ICI应用后T淋巴细胞浸润冠状动脉内皮和脂质斑块，增加斑块不稳定性，这可能是ICI应用后出现心肌缺血的病理生理基础。

### 1.5 瓣膜病变

临床上有个案报道用药后出现中-重度主动脉瓣、二尖瓣和三尖瓣关闭不全，且多数同时合并心肌病变（心肌炎或心肌病）。病理生理基础推测可能与T细胞浸润瓣膜产生炎性改变、纤维化以及由于心肌病变造成瓣膜附属器功能失调有关^[10]^。

### 1.6 其他

有报道部分ICI使用患者会出现血压升高，但具体发生率不详。此外，ICI会导致血管炎，主要表现为颞动脉炎和风湿性多肌痛，这两类疾病的诊疗会在ICI风湿病相关副作用的文章中介绍。

## 2 ICI心脏副作用出现的特征

研究发现，上述ICI相关心脏副作用如心肌炎、心包疾病同时出现的几率不高。PD-1/PD-L1抑制剂比CTLA-4抑制剂似乎更易出现心肌炎或心包疾病，而CTLA-4抑制剂相对更多出现血管炎如颞动脉炎。PD-1/PD-L1抑制剂的心肌炎副作用可能与起始较高剂量（如纳武利尤单抗≥3 mg/kg）有关。两种ICI的联合使用会增加心肌炎的发生率，联合治疗一旦出现心血管副作用其死亡率也明显高于单药治疗（66% *vs* 44%）^[9]^。目标肿瘤方面，目前应用较多的是肺癌和恶性黑色素瘤，观察到肺癌患者似乎更容易出现心肌炎和心包受累，而恶性黑色素瘤患者更多出现心肌炎或血管炎^[9]^。各种致命性的心脏副作用可能与其他系统性损害同时出现，常见合并的系统损害为神经肌肉型损害（如骨骼肌肌炎、重症肌无力和格林巴利综合征）^[11, 12]^。所以如果应用ICI的患者出现骨骼肌乏力、疼痛等情况，或抽血发现肌酸激酶明显升高，考虑神经骨骼肌副作用的同时，要筛查心肌损害的相关指标如肌钙蛋白、BNP、心电图等，以免漏诊^[4]^。

## 3 ICI的心脏副作用的诊断流程

如上所述，尽管ICI发生心脏副作用的几率较低，一旦发生病程凶险，所以应时刻提高警惕，早期发现心脏异常。一旦出现胸闷、胸痛等临床症状，应尽快完成炎症指标（血沉、C反应蛋白）、心肌酶、BNP、心电图、超声心动图等无创评估，应注意动态观察上述检测指标以评估病情严重程度同时进行病因的鉴别诊断。一旦发生心力衰竭，如果临床不能除外冠状动脉缺血的情况，应完善冠状动脉造影或冠状动脉增强CT。除外冠状动脉阻塞性病变后考虑心肌病变，需尽早完善增强心脏核磁共振；如患者合并明显的血流动力学异常或新发严重室性心律失常、Ⅱ度及以上房室传导阻滞等情况，在给予充分支持治疗同时有条件时应行心内膜活检进一步明确诊断^[7]^，见图 1。

**1:**
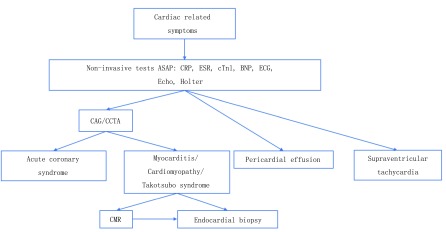
ICI相关心脏副作用的诊断流程图 Diagnostic flow chart of ICI-related cardiac side effects. ASAP: As soon as possible; CRP: C reactive protein; ESR: erythrocyte sedimentation rate; cTnI:cardiac troponin I; BNP: brain natriuretic peptide; ECG: electrocardiogram; Echo: chocardiography; CAG: coronary angiography; CCTA: coronary computed tomography angiography; CMR: cardiac magnetic resonance.

## 4 ICI心脏损害的处理

由于ICI严重心脏损害的发生率较低，目前的治疗建议主要基于个案报道和类似疾病的诊疗经验，尚无临床试验依据。2019年美国国立综合癌症网络（National Comprehensive Cancer Network, NCCN）关于免疫治疗相关毒性作用的指南中^[13]^，对于心脏毒副作用（主要针对急性心肌炎）建议完善检查后将病情严重程度分层。病情严重患者（有心律失常、心肌标志物和超声心动图异常，但血流动力学稳定）和危及生命（出现恶性心律失常、严重心肌病和血流动力学异常）的患者，均需要立刻停用ICI治疗，尽早给予甲基强的松龙每日1 g冲击治疗，持续3 d-5 d直到病情开始好转后减量。危及生命患者给予甲基强的松龙冲击治疗后如24 h内病情无缓解迹象，可加用人免疫球蛋白以及抗人胸腺免疫球蛋白或英夫利西单抗治疗，而近期也有个案报道提示血浆置换或其他一些抵抗炎症因子的生物制剂可能有效^[14-16]^。如果患者出现持续顽固性心力衰竭，除血管活性药物外，可以尝试动脉内球囊反搏或体外膜肺氧合支持治疗^[17]^。除心肌炎外，表 1对其他ICI相关心脏副作用的处理进行总结。

**1:** ICI常见心脏副反应处理建议 Recommendations for the management of common cardiac side effects in ICI

	Immunosuppressants	Therapy for heart disease
Confirmed myocarditis	Methylprednisolone 500 mg-1, 000 mg daily and intravenous immunoglobulin until clinically stable, followed by oral prednisolone 1 mg/kg once daily; If not stable, infliximab/anti-thymocyte globulin/abatacept/plasmapheresis	Diuretics, ACE inhibitor; *β* blocker if sinus tachycardia, atrial tachycardia, ventricular tachycardia, or ventricular fibrillation
New left ventricular systolic dysfunction without inflammation	No	According to the heart failure guideline (diuretics, ACEI, *β*-bloker *et al*)
Takotsubo syndrome	No	diuretics, ACEI, *β*-bloker
Ventricular tachycardia or ventricular fibrillation	Intravenous methylprednisolone 500 mg-1, 000 mg daily if myocarditis evident until clinically stable and troponin-negative, followed by oral prednisolone 1 mg/kg once daily	Emergency defibrillation; If hemodynamic stable, consider amiodarone or lidocaine or *β* blocker
New advanced conduction disease (second-degree or third-degree heart block)	Consider intravenous methylprednisolone if coexisting myocarditis	Emergency pacing
New atrial fibrillation/flutter (Exclude myocarditis)	No	Closely observe troponin，BNP, ECG, Echo; Find underlying disease: hypoxia, infection, thyroid dysfunction *et al*. Evaluate rhythm control or rate control. Consider anticoagulation unless contradiction.
Pericarditis with/without cardiac tamponade	Consider intravenous methylprednisolone 500 mg-1, 000 mg daily until clinically stable.	Emergency pericardiocentesis if needed. Consider NSAIDs
Acute myocardial infarction	If consider coronary arteritis, intravenous methylprednisolone is indicated	Emergency coronary angiography. Antiplatelet, statin, ACE inhibitor; *β* blocker
New asymptomatic increase in cardiac troponin	No	Closely observe troponin，BNP, ECG, Echo
Supraventricular tachycardia	No	Closely observe troponin，BNP, ECG, Echo; Find underlying disease: hypoxia, infection, thyroid dysfunction *et al*.
ECG：electrocardiogram；Echo：echocardiography；BNP：brain natriuretic peptide；NSAIDs: Non-Steroidal Antiinflammatory Drugs.		

## 5 识别ICI心脏副作用的高危人群

目前尚无对ICI心脏副作用高危人群的高质量研究，但是下述人群可能需要格外关注。首先，联合应用ICI药物或应用ICI药物同时合并使用其他心脏毒性药物（如VEGF酪氨酸激酶抑制剂）的患者；其次，有使用蒽环类药物或其他心脏毒性药物病史的患者；另外，出现ICI药物其他系统相关毒性尤其是骨骼肌和神经系统病变时要特别注意是否合并心脏副作用。既往患有自身免疫性疾病（如系统性红斑狼疮、类风湿关节炎、结节病），有基础心脏疾病（如冠心病、心力衰竭、心肌炎、化疗后心力衰竭病史）的患者也需特别注意。

## 6 ICI心脏毒性的检测和早期发现

鉴于ICI相关心脏毒性的高致死率，密切监测临床病情变化，早期发现病情改变对改善患者预后尤为重要。用药前，应注意询问并记录患者心脏病史，识别高危患者，同时完善基线化验检查（包括cTnI、NT-proBNP/BNP、心电图、超声心动图、炎性指标），如有可疑心肌缺血症状，需完善心肌负荷试验或冠脉CTA；如有心律失常，需完善24 h动态心电图^[18]^。

治疗过程中的监测：每次随诊需仔细询问患者心脏相关症状，查体并记录心率、血压、心脏杂音、心包摩擦音、肺内啰音、下肢水肿、颈静脉充盈情况；完善实验室检查包括cTnI、NT-proBNP、ECG、Echo、胸片；在用药前6周上述指标需每1-2周监测一次，如指标有新发异常应动态观察，并咨询心脏科医生^[19]^。

## 7 总结

ICI在恶性肿瘤治疗中的应用给肿瘤科医师和患者带来了新的希望。这类药物心脏副作用的发生率较低，但是致死性高，多为急性发病；建议用药前完善心血管相关检查，识别高危患者，用药期间严密监测心血管相关指标，一旦可疑心脏损伤，应抓紧时间快速明确诊断，以早期治疗，改善预后。
